# Differentiating Cell Entry Potentials of SARS-CoV-2 Omicron Subvariants on Human Lung Epithelium Cells

**DOI:** 10.3390/v16030391

**Published:** 2024-03-01

**Authors:** Revansiddha H. Katte, Yuanyun Ao, Wang Xu, Yang Han, Guohua Zhong, Dibya Ghimire, Jon Florence, Torry A. Tucker, Maolin Lu

**Affiliations:** Department of Cellular and Molecular Biology, School of Medicine, University of Texas at Tyler Health Science Center, Tyler, TX 75708, USA; revansiddha.katte@uttyler.edu (R.H.K.); yang.han@uttyler.edu (Y.H.); torry.tucker@uttyler.edu (T.A.T.)

**Keywords:** Omicron XBB, Omicron subvariants, spike protein, virus-cell entry, fusion/fusogenicity, BEAS-2B, human lung epithelium

## Abstract

The surface spike (S) glycoprotein mediates cell entry of SARS-CoV-2 into the host through fusion at the plasma membrane or endocytosis. Omicron lineages/sublineages have acquired extensive mutations in S to gain transmissibility advantages and altered antigenicity. The fusogenicity, antigenicity, and evasion of Omicron subvariants have been extensively investigated at unprecedented speed to align with the mutation rate of S. Cells that overexpress receptors/cofactors are mostly used as hosts to amplify infection sensitivity to tested variants. However, systematic cell entry comparisons of most prior dominant Omicron subvariants using human lung epithelium cells are yet to be well-studied. Here, with human bronchial epithelium BEAS-2B cells as the host, we compared single-round virus-to-cell entry and cell-to-cell fusion of Omicron BA.1, BA.5, BQ.1.1, CH.1.1, XBB.1.5, and XBB.1.16 based upon split NanoLuc fusion readout assays and the S-pseudotyped lentivirus system. Virus-to-cell entry of tested S variants exhibited cell-type dependence. The parental Omicron BA.1 required more time to develop full entry to HEK293T-ACE2-TMPRSS2 than BEAS-2B cells. Compared to unchanged P681, S-cleavage constructs of P681H/R did not have any noticeable advantages in cell entry. Omicron BA.1 and its descendants entered BEAS-2B cells more efficiently than D614G, and it was slightly less or comparable to that of Delta. Serine protease-pretreated Omicron subvariants enhanced virus-to-cell entry in a dose-dependent manner, suggesting fusion at the plasma membrane persists as a productive cell entry route. Spike-mediated cell-to-cell fusion and total S1/S2 processing of Omicron descendants were similar. Our results indicate no obvious entry or fusion advantages of recent Omicron descendants over preceding variants since Delta, thus supporting immune evasion conferred by antigenicity shifts due to altered S sequences as probably the primary viral fitness driver.

## 1. Introduction

**Figure 1:**
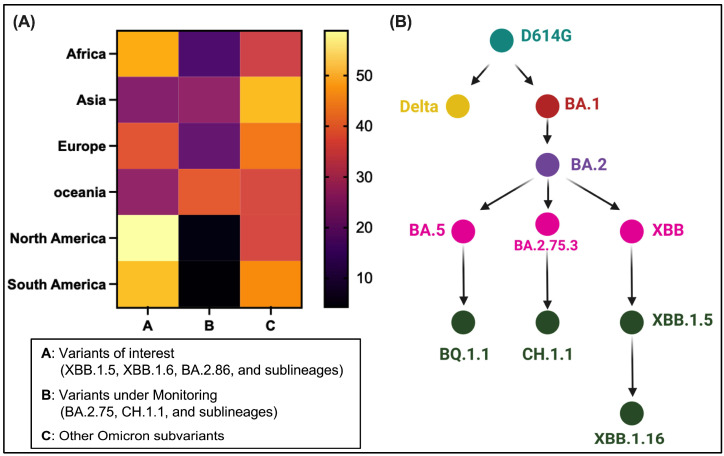
Geographical prevalence and evolution of emerged SARS-CoV-2 Omicron and its subvariants (**A**) Geographical prevalence until January 2024 (data source: GISAID) of circulating Omicron subvariants. The heat map indicates the cross-continental relative circulating frequencies of subgroups of omicron subvariants, including those of interest, under monitoring, and others. The color bar shows the percentages of circulating variants. (**B**) Evolution and origin of Omicron descendants, XBB.1.5 and XBB.1.16 variants. BA.1 is the parental Omicron that outcompeted the preceding Delta variant. Both XBB.1.5 and XBB.1.16 are descendants of the BA.2 subvariant, XBB.

**Figure 2:**
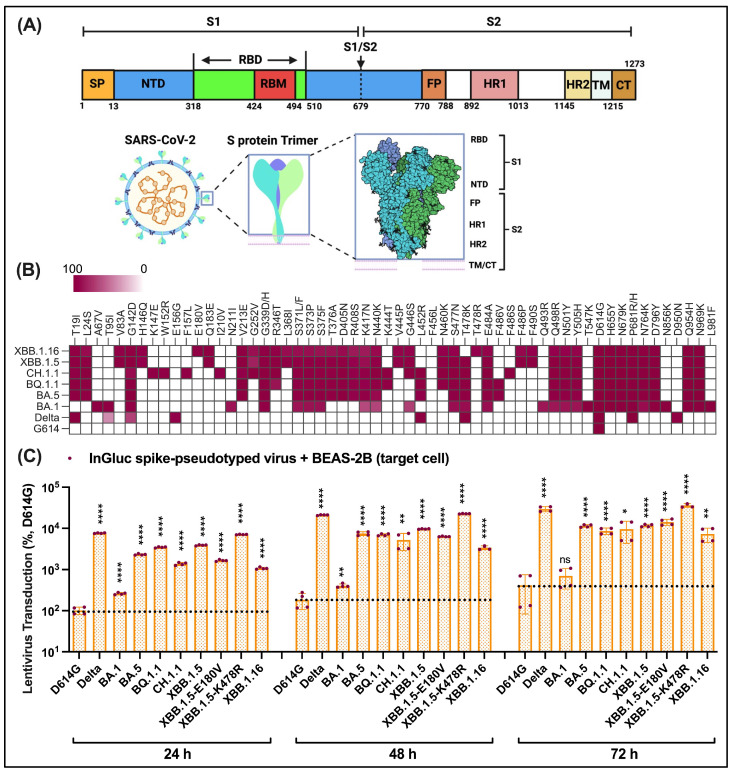
Sequence diversity of Omicron variants and pseudovirus transduction comparisons of Omicron lentiviruses on BEAS-2B lung epithelium cells. (**A**) Domain organization of SARS-CoV-2 surface spike (S) glycoprotein. S1/S2, furin cleavage sites; SP, signal peptide; NTD, N-terminal domain; RDB, receptor-binding domain; RBM, receptor-binding motif; FP, fusion peptide; HR1/HR2, heptad repeat 1/2; TM, transmembrane domain; CT, cytoplasmic tail. (**B**) Heat map indicating the frequency of acquisition of substitutions in S of circulating Omicron variants. (**C**) Time course infectivity comparisons of Omicron lentivirus on BEAS-2B bronchial epithelium cells in comparison to those of D614G and Delta. S pseudovirus transduction was evaluated based on a Gaussia luciferase reporter gene packaged into S lentiviruses and tittered on BEAS-2B bronchial epithelium cells. Results (mean ± SD) were normalized and compared to D614G measured after 24 h post-infection. The black dashed lines indicate the comparison reference. Statistical significance was evaluated using a *t*-test (* *p* < 0.05; ** *p* < 0.01; **** *p* < 0.0001; ns, non-significant). Unless specified, Omicron lentiviruses here include lentiviruses carrying individual BA.1, BA.5, BQ.1.1, XBB.1.5, XBB.1.5-E180V, XBB.1.5-K478R, and XBB.1.16 S variants.

Severe acute respiratory syndrome coronavirus 2 (SARS-CoV-2), the causative pathogen of the devastating coronavirus disease 2019 (COVID-19) pandemic, has burdened global public health, thus necessitating continuous surveillance of virus infection waves in the future [1,2,3]. Since the COVID-19 outbreak, thousands of SARS-CoV-2 variants have emerged, with the most recent being the dominant Omicron subvariants (Figure 1), and have continuously challenged administrated controlling measures [4,5]. As of December 2023, COVID-19 has claimed approximately 7.0 million lives with a confirmed caseload topping 772.4 million worldwide. The historically fastest developed vaccines and monoclonal antibody therapies were implemented in 2020 and have effectively saved millions of lives since their development. These interventions target highly dynamic virus surface proteins known as spikes (S) (Figure 2A,B) [6,7,8,9,10,11,12,13], which are the primary targets of antibody responses [14,15,16,17,18,19,20,21].

The spike protein drives the entry of SARS-CoV-2 into susceptible cells by mediating fusion at the plasma membrane or endocytic pathway [7,22,23,24,25,26]. S is initially synthesized as a single-chain polypeptide precursor and trimerized into a spike precursor, which is later proteolytically cleaved into non-covalently linked (S1/S2)_3_ [7,13,22,23,24,25,26] (Figure 2A). The spike protein has two cleavage sites, one at S1/S2 and the other at S2′ [24,25,26,27]. S1/S2 is the furin cleavage site at arginine (R685) within polybasic RRAR (R682 to R685). The polybasic S1/S2 site is required for effective cleavage by furin [24,25]. A second cleavage at the S2′ site by cellular transmembrane protease serine 2 (TMPRSS2) at the cell surface or cathepsin in endosomal compartments releases a fusion peptide and triggers downstream fusion events [24,25,26,27]. S-mediated cell entry depends on cleavages at both S1/S2 and S2′ sites by proteases in most cell lines. The entry routes include TMPRSS2-dependent fusion at the cell surface and cathepsin-dependent acidic endocytosis [24,25,26,27]. Mechanically, the receptor-binding domain (RBD) in the S1 subunit recognizes and binds the cell receptor human angiotensin-converting enzyme 2 (hACE2) [25,28,29,30,31] (Figure 2A). Three domains (fusion peptide—FP and heptapeptide repeat 1/2—HR1/HR2) in S2 play significant roles in the cell entry of SARS-CoV-2 [7,21,32] (Figure 2A).

SARS-CoV-2 viruses are under selection pressure due to vaccination and natural infection. Viruses continue to evolve to improve viral fitness and evade immunity, which is primarily achieved by constantly acquiring genetic changes in S [8,11,33,34]. Variants that caused severe global outbreaks were designated as variants of concern (VOC). In early 2020, the more transmissible spike G614 strain (D614G) outcompeted the Wuhan strain (D614), and D614G has persisted in all following variants. Later, multiple VOCs harboring unique sets of mutations in S have emerged across the globe, including the Alpha, Beta, Gamma, Delta, and Omicron. Omicron was first identified in late 2021, referred to as BA.1, and quickly surpassed the preceding Delta. BA.1 and its descendants harbor an unusually large number of mutations (over 30) in S [11,35,36]. These mutations confer an advantageous increase in viral transmissibility and evasion of immunity elicited by natural infection and vaccination [33,37,38,39,40,41,42]. There are hundreds of subvariants of Omicron (BA.1) that have evolved, such as BA.2, BA.5, and XBB. XBB subvariants, especially the recently dominant XBB.1.5 and XBB.1.16 lineages, reached a high level of resistance to neutralization by antibodies [42,43,44], resulting in infection breakthroughs in the populations with waning immunity. These Omicron subvariants with escape mutations in S demonstrate the necessity of vaccine boosters with S immunogens covering new variants and broad-spectrum antiviral agents.

Acquired mutations in S cause changes in virus-to-cell entry and cell-to-cell fusion potentials associated with viral infection and transmissibility. The evaluation of S-dependent virus-to-cell entry in the presence of serially diluted antibodies/inhibitors is commonly used to reflect antibody/drug potency/evasion. Virus infection and neutralization of S variants have been extensively investigated, and most of the used target cells have been HEK293T-hACE2, HEK293T-ACE2-TMPRSS2, CaLu-3, Huh-7, and Vero-E6 [25,39,41,43,45,46,47,48,49,50,51]. In this study, we adapted a more physiologically relevant human bronchial epithelium cell line, BEAS-2B, as the target cell and advanced our previous split NanoLuc-based entry/fusion assay [9] using a spike pseudotyped lentivirus system. Based on a reconstituted fusion reporter in BEAS-2B cells, we systematically documented time course virus-to-cell entry and cell-to-cell fusion of Omicron XBB.1.5 and XBB.1.16 alongside preceding variants, including D614G, Delta, BA.1, BA.5, BQ.1.1, and CH.1.1. The evaluation of entry/fusion abilities of S variants using human airway epithelium cells and an advanced entry/fusion assay will enhance our understanding of SARS-CoV-2 evolutionary biology, offering implications for transmissibility advantages of Omicron subvariants.

## 2. Materials and Methods

### 2.1. Cell Lines Culture

HEK293T cells (human embryonic kidney, CRL-3216, ATCC) were used to prepare spike pseudotyped lentivirus. HEK293T-hACE2 (NR-52511, BEI), 293T-hACE2-TMPRSS2 (NR-55293, BEI), and HEK293T cells were cultured in high glucose Dulbecco’s modified Eagle medium (DMEM) (Gibco, Cat # 11-965-092, Waltham, MA, USA) supplemented with 10% (*v*/*v*) fetal bovine serum (R&D Systems, Cat # S11550, Minneapolis, MN, USA) and 1% penicillin-streptomycin (Gibco, Cat # 15140-122). BEAS-2B cells (CRL-3588, ATCC) were used as target cells to quantify spike lentivirus infectivity and fusogenicity. BEAS-2B cells were cultured in a bronchial epithelial growth medium (BEGM) containing a BEBMTM bronchial epithelial cell growth basal medium (Lonza, Cat # CC-3171) and BEBMTM bronchial epithelial cell growth medium SingleQuotsTM supplements and growth factors (Lonza, Cat # CC-4175), using a BEGM^TM^-2 BulletKitTM medium (Lonza, Cat # CC-3170). The flasks, dishes, or plates were pre-coated with a mixture of 0.01 mg/mL fibronectin (Sigma, Cat # FC010, St. Louis, MO, USA), 0.03 mg/mL bovine collagen type I (Advanced BioMatrix PureCol, Cat # 5005, Carlsbad, CA, USA), and 0.01 mg/mL bovine serum albumin (LAMPIRE, Cat # 7500854, Pipersville, PA, USA) dissolved in a BEBM basal medium (Lonza, Cat # CC-3171, Basel, Switzerland).

### 2.2. Plasmids

D614G, Delta, BA.1, BA.5, BQ.1.1, CH.1.1, XBB.1.5, and VSV-G genes were synthesized and cloned into a pcDNA3.1 vector at the restriction sites of KpnI and XbaI by GenScript Biotech. XBB.1.5-E180V, XBB.1.5-K478R, and XBB.1.16 plasmids were constructed via site-directed mutagenesis, based on the backbone of the XBB.1.5 plasmid.

### 2.3. Spike Pseudotyped Lentivirus Transduction through Gaussia Luciferase Reporter Assay

The SARS-CoV-2 spike pseudotyped lentivirus was produced by co-transfecting HEK293T cells with plasmids of pCMV-dR8.2, pcDNA3.1-S, and intro-regulated Gluc (HIV-1-inGluc) [52] at a ratio of 2:2:1 using a transfection reagent, polyethylenimine “Max” (PEI MAX, Polyscience, Cat # NC1014320). After 40 h (h) post-transfection, the produced virus in the cell supernatants was collected and filtered using 0.45 µm filters and then concentrated via ultracentrifugation at 25,000 rpm for 2 h. P24 levels of all tested spike pseudo-lentiviruses were evaluated using a lentivirus titer p24 ELISA Kit (GeneScript, Piscataway, NJ, USA) to ensure that an equal number of viral particles were added to target cells. Viruses were then added to BEAS-2B cells pre-seeded in 48-well plates. At 24, 48, and 72 h post-infection, 100 µL cell supernatants were added to 96-well plates for quantifying the relative light units (RLU) of the Gaussia luciferase reporter using a Gaussia luciferase flash assay kit (Thermo Fisher Scientific, Cat # 16158, Waltham, MA, USA). Relative viral infectivity (mean ± SD) was determined by normalizing RLU to the mean value of its control counterpart.

### 2.4. Single-Round Virus-to-Cell Entry

Spike-dependent virus-to-cell entry was determined using the split NanoLuc (HiBiT/LgBiT) [53] system, as previously described [9] and quantified using a Nano-Glo^®^ substrate kit (Promega, Cat # N1120, Madison, WI, USA). Spike lentiviruses were produced via the co-transfection of HEK-293T cells with a plasmid mix of 4 µg psPAX2, 2 µg pLL3.7, 2 µg CypA -HiBiT, and 4 µg pcDNA3.1-S using PEI. The viral cell supernatants were harvested after 40 h transfection, then filtered by 0.45 µm filters, and then ultracentrifuged at 25,000 rpm for 2 h. In the case of using trypsin, viruses were treated with trypsin at concentrations of 0, 50, and 150 µg/mL at 37 °C for 15 min, and the reactions were stopped by adding FBS at a final concentration of 10%. BEAS-2B cells were transfected with 10 µg PH-LgBiT plasmid and seeded onto 48-well plates. The virus (100 µL) was added to the BEAS-2B cells and cultured for 72 h. Supernatants (50 µL) were collected at 24, 48, and 72 h for quantifying luciferase activities.

### 2.5. Spike Protein Processing in Cells

After 40 h post-transfection of HEK293T cells with pCMV-dR8.2 and pcDNA3.1 S plasmids, cells were washed with DPBS buffer and then lysed in RIPA (Thermo Scientific, Cat # 89900) and Halt protease inhibitor (Thermo-scientific, Cat # PI78430) for 30 min on ice. Protein samples in cell lysates were loaded on a 4–12% BisTris gel and transferred to a PVDF membrane by semi-dry blotting. The PVDF membrane was blocked with 2% (*w*/*v*) bovine serum albumin (BSA, GOLDBIO, Cat # A-420-250, St. Louis, MO, USA) in TBST (TBS with 0.05% Tween 20) for 1 h at room temperature and then probed with anti-S1 (Sino Biological, Cat # 40591, Beijing, China), anti-S2 (Sino Biological, Cat # 40590), and anti-beta actin (Sigma-Aldrich, Cat # A2228) in 2% BSA/TBST overnight at 4 °C. Secondary antibodies of goat anti-rabbit IgG (H + L) (Invitrogen, Cat # 31460, Carlsbad, CA, USA) and rabbit anti-mouse IgG (H + L) (Invitrogen, Cat # PA1-28568) were added on the membrane and incubated at room temperature for 1 h. After incubation with primary and secondary antibodies, the membrane was washed three times for 20 min each with TBST. Immunoblots were imaged using ECL Immobilon UltraPlus western HRP substrate (Millipore, Cat # WBLULP-100, Burlington, MA, USA) and exposed on an Azure 300 chemiluminescent western blot imager. The quantification of band intensities was determined using Image J software v1.52.

### 2.6. Cell-to-Cell Fusion

Confluent HEK-293T cells (80%) cultured in 10 cm dishes were co-transfected with a mixture of 5 µg pcDNA3.1-S and 5 µg CypA-HiBiT using PEI. Meanwhile, 80% confluent BEAS-2B cells in 10 cm dishes were transfected with 10 µg PH-LgBiT plasmid. After 24 h transfection, HEK-293T cells were treated with accutase (for detaching cells) for 10 min at 37 °C. The detached HEK-293T and 24h-transfected BEAS-2B cells were then mixed at a 1:1 ratio and seeded onto 48-well plates. After 24 h coculture, 100 µL mixed-cell supernatants were transferred to 96-well plates for luciferase activity measurement.

### 2.7. Statistics and Reproducibility

All statistical analyses were performed with GraphPad Prism 10.1.2 software. An unpaired *t*-test was used to analyze continuous variable datasets. A *p*-value less than 0.05 was considered statistically significant.

## 3. Results

### 3.1. Dominance and Unique S Mutations of Omicron Descendants

In February 2023, a recombinant Omicron subvariant XBB derived from two BA.2 variants, BJ.1 and BM.1.1.1, emerged and quickly established their circulating dominance across the globe. Statistical data from GISAID until January 2024 indicate that XBB descendants, XBB.1.5, XBB.1.6, EG.5, and a recombinant BA.2.86 have emerged as the dominant variants across continents, especially in North and South America (Figure 1A). The prevalence of variants is dynamic as viruses continue to mutate. XBB.1.6 is derived from XBB.1.5, a sublineage of XBB (Figure 1B). The receptor binding domain (RBD) and N-terminal domain (NTD) in the surface subunit S1 of S are regions of most accumulated mutations (Figure 2A,B). The XBB.1.6 S protein acquired new mutations, including E180V in the NTD and T478R in the RBD, whereas XBB.1.5 and the preceding CH.1.1, BQ.1.1, BA.5, BA.1, and Delta all harbored T478K (Figure 2B). XBB.1.16 inherited V83A, Q183E, G252V, L368R, V445P, F486P, and F490S substitutions from XBB.1.5. The fusion cleavage mutation P681R/H has been preserved ever since being adopted to the Alpha variant. Amino acid sequence comparisons of S variants (Figure 2B) were derived from genomic data deposited to GISAID. The diversity of genomic/amino acid sequences of prevalent S variants (Figure 1 and Figure 2) underlines the significance of exploring and understanding the biology of Omicron S subvariants compared with their preceding variants for updating COVID-19 vaccines and other antiviral agents.

### 3.2. Elevated Pseudovirus Transduction of Omicron Subvariants Compared to Prototype D614G but Reduced Compared to Delta

We first evaluated the transduction of lentiviruses pseudotyped with D614G, Delta, and Omicron, BA.1, BA.5, BQ.1.1, CH.1.1, XBB.1.5, and XBB.1.6, on the human bronchial epithelium cell, BEAS-2B, using our previously established Gaussia luciferase reporter assay [9,52] (Figure 2C). Delta and Omicron exhibited an apparent increase in infectivity at 24 h post-infection—77.58 times higher for Delta and 2.58 to 39.41 times higher for Omicron when compared to the prototype D614G. The infectivity of XBB.1.6, XBB.1.5, BA.5, BQ.1.1, and CH.1.1 was 4.31 to 15.25 times higher than the parent Omicron BA.1, of which XBB.1.5 was the highest among these variants. The infectivity of XBB.1.5 with the K478R mutation was 1.80 times higher than XBB.1.5, while XBB.1.5 with the E180V mutation showed slightly lower infectivity than XBB.1.5. The infectivity of XBB.16 dropped by 0.19 to 2.54 times relative to XBB.1.5, BA.5, BQ.1.1, and CH.1.1. We also showed the time-increased infectivity of these variants in BEAS-2B cells from 24 to 72 h. Consequently, these results suggested that XBB.1.5 and XBB.1.6 have acquired increased infectivity compared with D614G and Omicron BA.1.

### 3.3. Suboptimal Use of HEK293T-hACE2 in Quantifying SARS-CoV-2 Cell Entry

**Figure 3:**
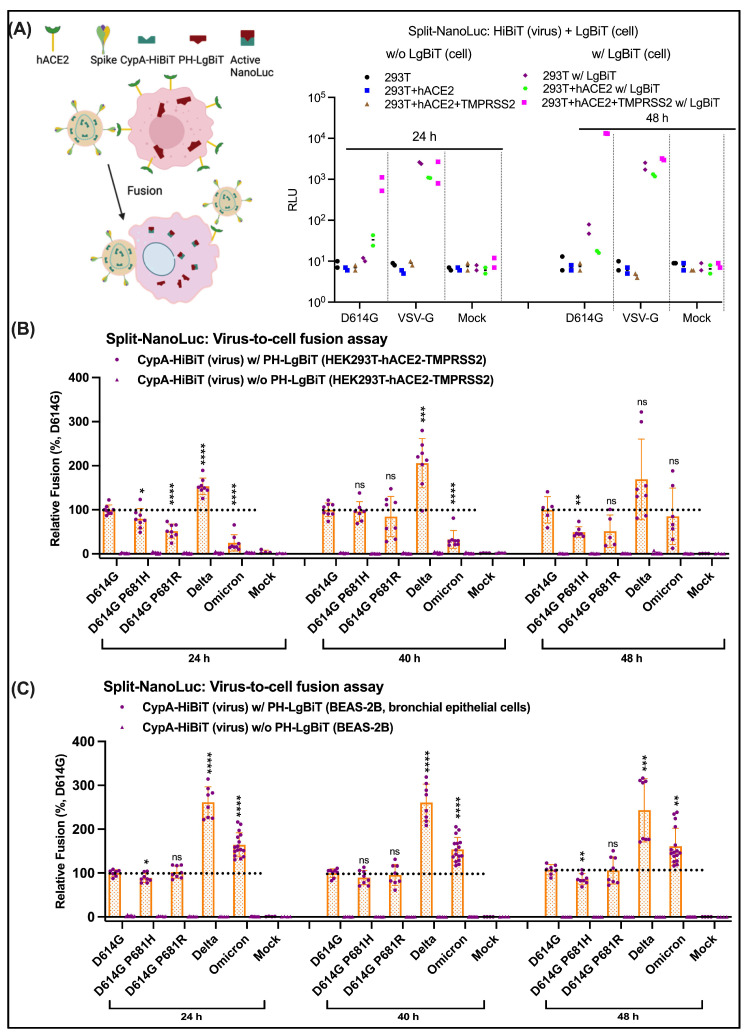
Acquired mutations at the S1/S2 cleavage site have no obvious effect on the entry of S lentiviruses into BEAS-2B lung epithelial cells using a clean split NanoLuc assay (**A**) Establishment and demonstration of a clean split NanoLuc fusion/entry readout assay by using D614G S vs. VSV-G pseudotyped lentiviruses. Only completion of HiBiT and LgBiT after cell entry exhibited luciferase activity, whereas lack of HiBiT or LgBiT resulted in no luciferase signal as negative controls. VSV-G lentivirus enters all three tested cell lines (HEK293T, HEK293T-hACE2, and HEK293T-hACE2-TMPSS2) as positive controls. D614G pseudotyped lentivirus demonstrated efficient entry into EK293T-hACE2-TMPSS2 cells. (**B**) Cell entry of D614G, P681H, P681R, Delta, and Omicron (BA.1) lentiviruses were evaluated using a split NanoLuc assay over time. With HEK293T-hACE2-TMPRSS2 as the target cell line, P681H/R point mutation at the S1/S2 cleavage site reduced entry potential of lentiviruses, and Omicron lentiviruses showed lagged entry established after 48 h post-infection. (**C**) Experiments as in (**B**) with BEAS-2B as the target cells. P681H/R at S1/S2 has no noticeable effect on lentiviruses’ entry into BEAS-2B lung epithelium cells. Effective entry of tested lentiviruses was detected after 24 h post-infection. The black dashed lines indicate the comparison reference. Statistical significance was evaluated using a *t*-test (* *p* < 0.05; ** *p* < 0.01; *** *p* < 0.001; **** *p* < 0.0001; ns, non-significant).

To validate our pseudovirus transduction results with a different assay, we adapted the split NanoLuc virus-to-cell assay to BEAS-2B cells. We then tested the entry potential of Omicron and its descendant variants. S lentiviruses were packaged with HiBiT, and BEAS-2B cells were transfected with LgBiT. The quantitative entry of a HiBiT-packaged S lentivirus into LgBiT expressing live BEAS-2B cells could be evaluated by measuring the luciferase activities of reconstituted HiBiT-LgBiT in the presence of its substrate (Figure 3). This assay is based upon active NanoLuc reconstituted from LgBiT and HiBiT. Successful virus-to-cell entry restores NanoLuc luciferase activity between lentiviruses carrying HiBiT and cells expressing LgBiT [9,53] (Figure 3A). It is a clean entry readout assay with an extremely low background, as demonstrated in the near-zero baseline signals from negative controls of cells lacking LgBiT and tens of thousands-fold increases in signals from positive controls of VSV-G pseudotyped viruses (Figure 3A). The D614G lentivirus was unable to enter HEK293T-hACE2 in contrast to its efficient entry into HEK293T-hACE2-TMPRSS2 cells. The result of TMPRSS2 utilization suggests that membrane fusion at the plasma membrane is the primary entry route for the D614G variant, and TMPRSS2 is essential in this process. Our result also implies that HEK293T-hACE2 lacking TMPRSS2 is not an optimal target cell line for studying SARS-CoV-2 virus-to-cell entry, at least for early variants, and the use of different cell lines may give different readouts.

### 3.4. Entry Affected by P681H/R at Cleavage Sites Is Cell-Type Dependent

We then compared virus-to-cell entry abilities of D614G, P681R, P681H, Delta, and Omicron (BA.1) lentiviruses on HEK293T-hACE2-TMPRSS2 (Figure 3B) and BEAS-2B lung epithelium cells (Figure 3C) using the above-established split NanoLuc reporter assay. We obtained different results when we tested on each cell line. A reduction in the entry/fusion ability of P681R and P681H was observed on HEK293T-hACE2-TMPRSS2 after 24 h and 48 h post-infection (Figure 3B). The cell entry potential of Omicron was barely detectable on HEK293T-hACE2-TMPRSS cells after 24 h post-infection and rose above D614G but below Delta after 40 h post-infection. In contrast, reconstituted NanoLuc activities of P681R, P681H, and Omicron were well-established on BEAS-2B cells after 24 h post-infection and remained unchanged after 40 h and 48 h post-infection (Figure 3C). Delta lentivirus exhibited the highest NanoLuc readout on both cell lines at different time points (Figure 3B,C). HEK293T-hACE2-TMPRSS2 cells overly expressed hACE2 and TMPRSS2 on the cell surface, which is likely supraphysiological. As such, the cell entry potential of SARS-CoV-2 can likely be more faithfully recapitulated by using human lung epithelium BEAS-2B cells. The results we obtained using BEAS-2B cells indicate that P681R/H has no apparent effect on virus-to-cell entry. Based on the observed discrepancy in entry abilities between tested S lentiviruses on HEK293T-hACE2-TMPRSS2 and BEAS-2B cells, more physiologically relevant cell lines that naturally express hACE2 and TMPRSS2 as target cells should be used in the evaluation of SARS-CoV-2 virus-to-cell entry.

### 3.5. Efficient Virus-to-Cell Entry of Omicron Descendants on BEAS-2B Cells

**Figure 4:**
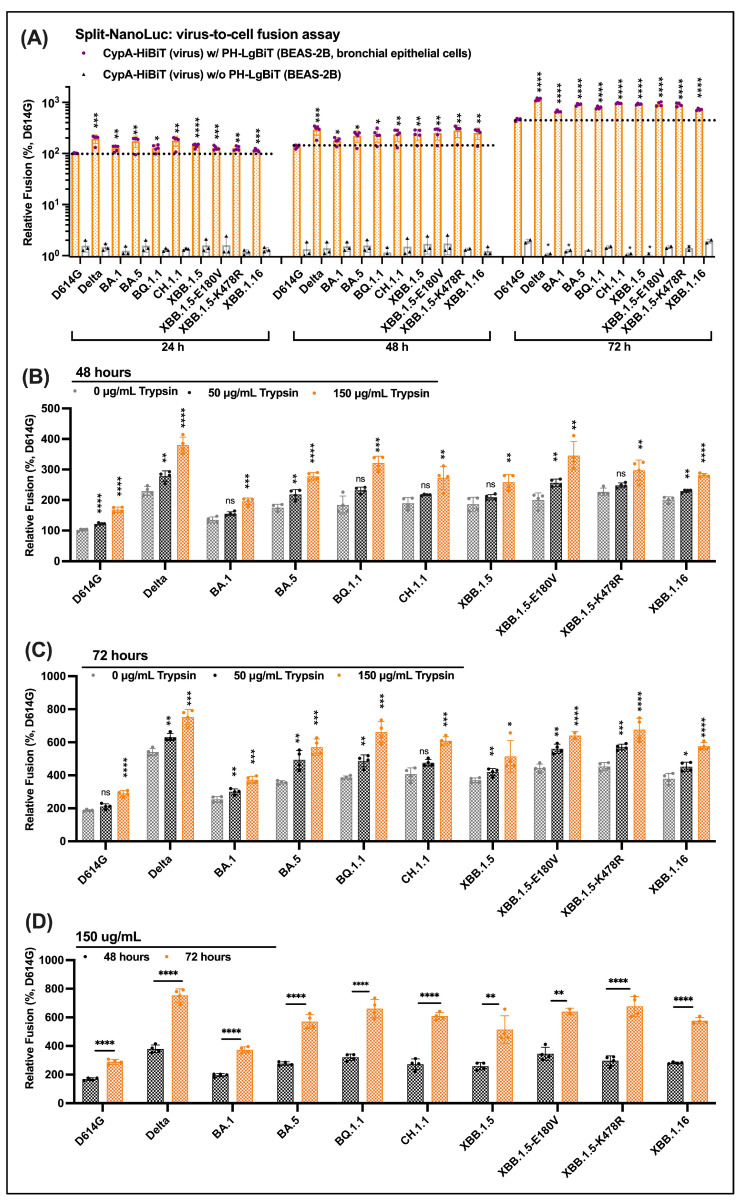
Omicron lentiviruses on BEAS-2B cells show efficient virus-to-cell entry that can be enhanced by serine protease (**A**) Time course measurements of entry efficiency of Omicron lentiviruses tittered on BEAS-2B cells using a split NanoLuc (HiBiT + LgBiT peptide fragments) fusion readout assay. HiBiT was packaged in spike lentiviruses, and LgBiT was expressed in BEAS-2B cells. Virus-to-cell entry was quantified based on the luciferase activities of reconstituted HiBiT-LgBiT in the presence of its substrate. Relative fusion (mean ± SD) was normalized to that of D614G measured after 24 h post-infection. (**B**–**D**) The promoting role of trypsin in regulating cell entry of S lentiviruses on BEAS-2B cells. Different doses of serine protease trypsin at indicated concentrations were added to lentiviruses before infecting the target BEAS-2B cells. Measurements were performed at different time points and compared, including 48 h post-treatment (**B**), 72 h post-treatment (**C**), and the comparison (**D**). Relative entry efficiency (mean ± SD) was normalized to that of D614G measured. The black dashed lines indicate the comparison reference. Statistical significance was evaluated using a *t*-test (* *p* < 0.05; ** *p* < 0.01; *** *p* < 0.001; **** *p* < 0.0001; ns, non-significant).

We adapted the split NanoLuc virus-to-cell assay to BEAS-2B cells. Using this assay, we tested the entry potential of Omicron and its descendent variants. We observed a clear increase in the virus-to-cell entry of Delta and Omicron variants as compared with D614G (Figure 4A), consistent with results of virus infectivity using inGluc. SARS-CoV-2 Delta pseudoviral demonstrated its highest virus-to-cell entry among these variants in our Gaussia luciferase and split NanoLuc reporter assays. Unlike the relatively low infectivity of Omicron BA.1 lentivirus observed using InGluc, split NanoLuc measurements showed efficient fusion of Omicron BA.1 into BEAS-2B cells after 24 h post-infection. The most recent XBB variants, including XBB.1.5 and XBB.1.16, exhibited a modest advantage in virus-to-cell entry compared with BA.1 and BA.5, and a somewhat slight decrease as relative to BQ.1.1 and CH.1.1 (Figure 4A). Individual E180V and K478R substitutions slightly elevated virus-to-cell entry compared with XBB.1.5, but in combination did not favor entry, implying no entry synergy of these two acquired mutations.

### 3.6. Serine Protease Trypsin Promotes Virus-to-Cell Entry of Delta, Omicron and Its Descendants

The serine protease TMPRSS2 proteolytically processes S2 protein at S2′ to enable virus-to-cell membrane fusion in a pH-independent entry pathway [25,54], and whose functions could be mimicked by the serine protease trypsin in vitro. Thus, we explored the potential roles of trypsin in virus-to-cell fusion and evaluated the effects of trypsin pre-treatment of Omicron lentiviruses on BEAS-2B entry using our split NanoLuc assay. S lentiviruses were treated with trypsin at the concentrations of 0, 50, and 150 µg/mL and were protease-inactivated before being added to BEAS-2B cells. All tested variants, including D614G, Delta, Omicron BA.1, BA.5, BQ.1.1, CH.1.1, XBB.1.5, XB.1.5, XBB.1.15-E180V, XBB.1.15-K478R, and XBB.1.16, exhibited a similar dose-dependent increase of virus-to-cell entry (Figure 4B–D). For all tested variants, viruses treated with higher doses of trypsin showed more efficient virus-to-cell fusion than lower or no doses. Virus-to-cell entry readout levels increased from 48 to 72 h post-infection. Our results imply that serine protease promotes virus-to-cell membrane fusion of D614G, Delta, Omicron, and its once-dominant descendants. On the basis of TMPRSS2 utilization, it seems that fusion at the plasma membrane remains a productive entry pathway of Omicron variants into lung epithelium cells. It is of note that our assays of serine protease utilization cannot directly conclude the fusion routes.

### 3.7. No Obvious Difference in Cell-to-Cell Fusion Mediated by Omicron S Descendants

**Figure 5:**
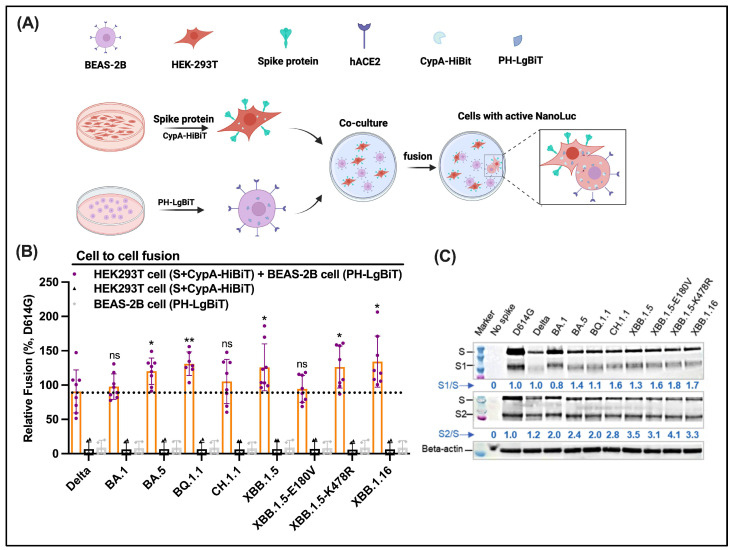
Enhanced or comparable spike-mediated cell-to-cell fusion and S1/S2 processing of Omicron descendants (**A**) Schematic illustrating the design of cell-to-cell fusion. HEK293T that carries S and CypA-HiBiT are co-cultured with BEAS-2B that carries PH-LgBiT. Successful cell-to-cell fusion as a result of S-hACE2 interactions give rise to the completion of CypA-HiBiT and PH-LgBiT—active NanoLuc, activity of which is measured in the presence of substrate to reflect fusion activities. (**B**) Spike-mediated cell-to-cell fusion of Delta, Omicron, and Omicron descendants alongside referenced D614G. Fusion is quantified by reconstituted NanoLuc (CypA-HiBiT + PH-LgBiT) signals, as illustrated in A. The black dashed lines indicate the comparison reference. Statistical significance was evaluated using a *t*-test (* *p* < 0.05; ** *p* < 0.01; ns, non-significant). (**C**) Assessment of total S1/S2 proteolytic processing in virus production cells using SDS-PAGE western blotting. Full-length S and S1 bands (top panel) were detected by an anti-S1 primary antibody. S and S2 bands (middle panel) were detected by an anti-S2 primary antibody. The ratio of S1/S or S2/S was calculated based on band intensities extracted using Image J software v1.52.

Next, we examined cell-to-cell fusion mediated by Omicron descendants. To compare the efficiency of cell-to-cell fusion, we co-transfected HEK-293T cells with plasmids expressing S and HiBiT and later cocultured them with target BEAS-2B cells transfected with LgBiT (Figure 5A). At 24 h post-coculture, cell-to-cell fusion was quantified by reconstituted NanoLuc readouts. Delta showed a similar level of cell-to-cell fusion compared with BA.1 (Figure 5B), which is different from its highest virus-to-cell fusion (Figure 3 and Figure 4). In contrast, BA.1, BA.5, BQ.1.1, XBB.1.5, XBB.1.5-E180V, XBB.1.5-K478R, and XBB.1.6 exhibited comparable cell-to-cell fusion in comparison with Delta (Figure 5B). As a control, we extracted membrane protein from HEK293T cells using Mem-PER™ Plus Membrane Protein Extraction Kit (Fisher Scientific, Cat#89842) and evaluated the S1 and S2 levels using immunoblotting. The results of Omicron subvariants were similar. The similarity of S1 and S2 levels on the gel indicates that the amount of S protein on the plasma membrane is equivalent, further indicating that the results of cell-to-cell fusion represent the fusion ability of S Omicron subvariants on BEAS-2B cells.

### 3.8. Omicron Subvariants Total S1/S2 Proteolytic Processing in Cells

Next, western blotting was performed to evaluate S proteolytic processing/cleavage at S1/S2 sites in virus producing HEK-293T cells (Figure 5C). Compared with D614G, these Omicron variants showed the increased S1/S2 cleavage of S proteins based on the results of the detected S1/S and S2/S ratios. No obvious increase in Delta S1/S2 proteolytic processing was detected. XBB.1.16 exhibited a comparable S processing to that of XBB.1.5. Both had a notably increased S processing compared with D614G and BA.1. A similar efficiency of S protein processing was observed for XBB.1.5-E180V and -K478R mutants as that of XBB.1.5. Since all Omicron descendants and Delta carry the P681R/H substitutions, the elevated S1/S2 processing is most likely irrelevant to P681R/H. Together, these results indicate that the Omicron descendants are more processed (at least not less) at S1/S2 relative to the preceding ones. Our S proteolytic processing results reflect the total level of S, including intracellular S and cell surface-expressed S. Our results do not necessarily reflect the processing efficiency of functional cell surface-expressed S that mediates cell-cell fusion. Nevertheless, the total S processing observed here is in accord with their cell-to-cell fusion abilities.

## 4. Discussion

SARS-CoV-2 mutates over time to improve viral fitness and evade immunity, which is primarily achieved by constantly acquiring genetic changes/amino acid substitutions in the vaccine and antibody target—the S protein [8,11,33]. Omicron BA.1 and its descendants carrying extensive S mutations have been the dominant circulating forms ever since it outcompeted Delta [3,4]. This study systematically compared multiple of the most recently emerged once-dominant Omicron subvariants regarding virus-to-cell entry and cell-to-cell fusion on bronchial epithelium cells. Our results provide insights into S cleavage, cell entry routes, and transmissibility of Omicron subvariants.

The comparison of different cell lines used in our fusion assays demonstrated the need to use human airway epithelium to correlate closely with in vivo and clinical implications. Besides real-time genetic monitoring of virus evolution, scientists have been racing against time to understand the up-to-date biology of S variants and implications for viral fitness in the context of virus transmissibility and immune evasion/antigenicity shifts due to altered S sequences [11,33,42,43,44]. In most laboratory settings, HEK293T-hACE2, HEK293T-ACE2-TMPRSS2, CaLu-3, Huh-7, A549, and Vero-E6 [25,39,41,43,45,46,47,48,49,50,51,55,56] have been used as host cells to study viral transmissibility and antibody evasion. These commonly used cell lines are relatively convenient to culture and maintain in virological and cellular laboratories. They are readily available for the timely evaluation of newly emerged S variants in response to the high mutation rate of S. Nevertheless, there are likely some drawbacks. For instance, cell lines overexpressing hACE2 or TMPRSS2 have the merit of manifesting or exponentially amplifying sensitivities for screening virus neutralization and inhibition sensitivities to antibodies and drugs. However, tumorigenic and non-human cell lines may have different response times and sensitivity to viruses from their respective non-cancerous cell lines and human cells. It is challenging to identify how accurately or faithfully the use of kidney HEK293T with overexpressed receptor/co-factor or lung cancerous CaLu-3, liver tumorigenic Huh7, non-human lung epithelium Vero-E6, or human lung adenocarcinoma A549 cell lines can recapitulate the real-world infection and transmission of SARS-CoV-2, which is primarily through epithelial cells in respiratory/airway tracts as the first gateway [33,57]. In this study, we advanced our previously used split NanoLuc-based fusion assay [9,53] and adapted human bronchial epithelium BEAS-2B in comparison to HEK293T as target cells for a spike pseudotyped virus infection. HEK293T-hACE2, in our study, did not show cell entry of D614G lentiviruses using a split NanoLuc fusion assay (Figure 3A). HEK293T-hACE2-TMPRSS2 that overexpresses the receptor-cofactor showed a slower response to Omicron BA.1 than D614G and Delta, whereas lung epithelium cells, BEAS-2B, with natural expression levels of the receptor-cofactor did not reveal that phenotype (Figure 3B,C). The observed differences underline the relevance of using human airway cells to study SARS-CoV-2 biology.

Our results on S1/S2 cleavage mutants and the use of BEAS-2B cells reveal a disassociation between furin cleavage and transmissibility. Cleavage-relevant mutations arise at P681 proximal to the S1/S2 polybasic site, such as P681H in Alpha and Omicron and P681R in Delta. Both arginine (R) and histidine (H) are basic (high pH) residues. P681R/H is expected to have viral fitness advantages considering its conservancy in all Omicron descendants. However, there is no consensus on whether and how P681R/H influences the ability of SARS-CoV-2 to enter or spread between cells [33,58], although evidence indicates the slight or significant enhancement of P681R/H in S1/S2 cleavage [59,60,61]. To avoid the potential effects of other mutations in S, we introduced P681R and P681H separately into D614G and did not observe any virus-to-cell entry advantages on both HEK293T-hACE2-TMPRSS2 (reduced level, Figure 2B) and BEAS-2B cells (comparable level, Figure 3C). The reduced cleavage observed on HEK293T-hACE2-TMPRSS2 cells is in line with the altered usage of TMPRSS2 by Omicron [62]. The findings of no entry advantages of furin cleavage mutations, however, challenge the results of directly associating high fusogenicity, transmissibility, and pathogenicity with enhanced spike cleavage conferred by P681H/R [59,60]. The functional consequences of S1/S2 furin cleavage mutations remain elusive, and it is likely additional mutations in S may be involved in enhancing virus transmissibility.

Our results of protease at S2’ cleavage revealed an additive effect on the entry route of SARS-CoV-2 variants to the cell surface. Early S variants, such as D614G (Figure 3A), showed strong entry dependence on TMPRSS2 [24,25,26,27], implying the primary entry route was through viral membrane fusion at the plasma membrane. In contrast, several studies indicated that Omicron alters the entry route towards the TMPRSS2-independent endocytic pathway, as evidenced in higher sensitivity to the cathepsin inhibitor than the TMPRSS2 inhibitor [45,62,63]. In this study, we observed that trypsin enhanced Omicron virus-to-cell entry on BEAS-2B cells in a dose-dependent manner, and the enhancement was conserved across all tested Omicron subvariants (Figure 4B). This finding suggests that the availability or abundance of expressed TMPRSS2 may be associated with the holding proportions of Omicron entry routes. When TMPRSS2 is abundantly expressed on airway cells, Omicron and its subvariants may still primarily use TMPRSS2-mediated entry, although it may be less efficient than cathepsin-mediated endosomal entry [45,62,63], which was not investigated in this study. Our results imply that entry at the plasma membrane remains a productive cell entry route for the most recently emerged Omicron subvariants. Our results are limited to BEAS-2B cells in the respiratory upper tract and lack the use of TMPRSS2 inhibitors. Future studies using the TMPRSS2 inhibitor camostat and the accurate evaluation of TMPRSS2 levels in different areas of respiratory tracts may offer new insights into the association between cell entry routes of Omicron subvariants and their transmissibility and pathogenicity.

The consolidation of virus-to-cell, cell-to-cell, and total S processing did not show obvious cell entry or fusogenicity advantages of Omicron subvariants over each other. Using the bronchial epithelium BEAS-2B cells, we showed that the virus-to-cell entry efficiency of Omicron BA.1 is higher than G614, but lower than the Delta S (Figure 3 and Figure 4), which supports the attenuated fusogenicity of Omicron reported in many studies [33,45,62,64]. In terms of cell-to-cell fusion and S processing, the differences between Delta, BA.1, and D614G were not apparent. Almost all tested Omicron descendants exhibited comparable levels of efficient single-round virus-to-cell entry, cell-to-cell fusion, and total S processing (Figure 4 and Figure 5). Collective observations suggest that dominant Omicron and its descendants have no obvious single-round entry or fusion advantages over preceding ones since Delta. Virus fitness involves multiple factors, including infectivity, transmissibility, antigenicity properties, host immune responses, pathogenicity, environmental factors, etc. Delta was a highly pathogenic and evasive variant but was surpassed by Omicron, although Omicron-infected individuals had mild clinical symptoms (less pathogenic than Delta) [4,65,66]. Consistent with our observations, strong evidence has suggested the lack of fusogenicity advantages in Omicron over preceding ones or Delta [33,45,62,64,66]. Hundreds of Omicron subvariants have evolved, and once-dominant descendants have demonstrated a higher ability to replicate, infect, and spread than the preceding ones. The transmissibility advantages of these once-dominant Omicron subvariants, such as XBB.1.5 and XBB.1.5, are likely primarily attributed to immune evasion associated with altered antigenicity properties due to the highly mutated rate of S functional regions [11,35,36,42,43,44,63]. Evasive properties may explain the high numbers of reinfections and vaccine breakthrough infections due to newly emerging Omicron subvariants.

Our current study is limited to the comparison of just one type of human bronchial airway epithelium (upper respiratory tract) with HEK293T-hACE2 and HEK293T-TMPRSS2 in our entry or fusion assays. Further investigations using multiple respiratory cell lines covering nasal and low respiratory tract epitheliums and even different organoid models in entry or fusion studies are required to produce more comprehensive insights into Omicron lineage/sublineage transmissibility advantages. Our study is also limited to using spike pseudotyped lentiviruses instead of authentic viruses, as a higher biosafety level is required for infectious viruses. Nevertheless, pseudotyped viruses are commonly used in studying SARS-CoV-2 biology, and BEAS-2B is a physiologically relevant cell line to SARS-CoV-2 infection. Therefore, our results should provide valuable insights into the clinically pertinent biology of Omicron subvariants.

## Data Availability

The data presented in this study are available on request from the corresponding author.
